# Effect of oral probiotics on postoperative nutrition level of patients with liver cancer

**DOI:** 10.1515/med-2026-1485

**Published:** 2026-08-12

**Authors:** Xiaohan Wang, Ziming Wang

**Affiliations:** Teaching and Research Section of Surgery, Henan Vocational College of Nursing, Anyang, China; First Department of Surgery, Anyang Cancer Hospital, Anyang, China

**Keywords:** liver cancer, probiotics, postoperative nutrition level, patient generated subjective global assessment score, arm muscle circumference

## Abstract

**Objectives:**

To determine the effect of probiotics on the postoperative nutritional status of patients with liver cancer.

**Methods:**

A total of 112 patients with liver cancer were enrolled from January 2021 to December 2022 and randomly assigned to either a probiotics group (n=56) or a placebo group (n=56). Patients in the probiotics group received oral administration of either bifid-triple viable capsules (420 mg, three times daily) or *Bacillus subtilis* capsules (0.5 g, three times daily) for 21 consecutive days. Patients in the placebo group received identical-looking placebo capsules following the same schedule. All patients received routine nursing care and nutritional support during hospitalization. Nutritional status was assessed at baseline (day 0) and at 7, 14, and 21 days after intervention using the patient-generated subjective global assessment (PG-SGA) score, serum levels of albumin (Alb), prealbumin (PA), hemoglobin (Hb) and transferrin (TRF), as well as arm muscle circumference (AMC) and triceps skinfold thickness (TSF) measurements.

**Results:**

After 21 d, PG-SGA score was obviously reduced in probiotics group and placebo group relative to 0 d (p*<*0.05). Moreover, the decrease of PG-SGA score was more in probiotics group than placebo group (p*<*0.05). After 21 d, the increased levels of Alb, PA, Hb and TRF in serums were more in probiotics group than placebo group (p*<*0.05). Furthermore, the increases of AMC value and TSF value were more in probiotics group than placebo group after 21 d (p*<*0.05).

**Conclusions:**

Oral probiotics had a good feedback on improving postoperative nutrition level in patients with liver cancer.

## Introduction

Liver cancer, one of malignant tumors, possesses significant impact on human life and has a high mortality at the late stage in the world [1], 2]. There are numerous risk factors for liver cancer, mainly including smoking, drinking, viral hepatitis, metabolic dysfunction and carcinogenic diet [3], [4], [5]. In the early stage, common clinic symptoms of liver cancer are loss of appetite, hidden pain in right upper abdomen, jaundice and unknown fever and edema. In the middle and late stage, some typical symptoms in liver cancer patients are emerged, such as intermittently persistent liver pain, continuous fever, severe jaundice, fatigue, emaciation and general weakness [6], 7]. In recent years, many therapies have been applied to treat liver cancer, such as hepatic resection, liver transplantation, chemotherapy, radiotherapy and immunotherapy [8], 9]. Yet, poor prognosis and easy recurrence are still problems for people with liver cancer and the 5-year survival rate is only 0–48 % [10]. Therefore, it is urgent to find more effective methods to improve poor prognosis in liver cancer patients.

Remarkably, recent studies have illustrated that postoperative nutrition level in liver cancer patients is closely linked with postoperative recovery, which is a new perspective to improve liver cancer disease [11], 12]. Previous documents have elucidated that cancer patients’ nutritional statuses are closely related to overall health statuses and potential tumor growth [13], 14]. The good therapeutic effect can be realized via effective anti-cancer therapy and preservation of the host status. Hence, postoperative nutrition level can potentially reflect the therapeutic effect of different therapy ways. Currently, some studies have described the effects of probiotics on postoperative infection prevention and antitumor [15], 16]. However, there is rare report about the role of probiotics in improving postoperative nutrition level in liver cancer patients, which is further explored in this trial.

Probiotics, belonging to one of enteral nutrition support substances, plays an important role in immunomodulation and is a preventive measure against liver cancer disease [17]. The main functions of probiotics are to promote the digestion and absorption of nutrients, improve the immunity, maintain the balance of intestinal flora and protect the intestinal mucosal barrier [18]. The common probiotics chiefly includes bifid-triple viable (including bifidobacterium, lactobacillus acidophilus and enterococcus faecalis) and bacillus subtilis, which have been used to improve nutritional status in clinic [19].

The rationale behind this study is that enhancing postoperative nutrition through probiotic supplementation may improve recovery and clinical outcomes in liver cancer patients. We hypothesize that oral probiotic administration can significantly improve the postoperative nutritional status of patients undergoing liver cancer surgery. Therefore, the present study aims to answer the following research question: Can oral probiotics effectively improve the postoperative nutritional status in patients with liver cancer? To address this, we conducted a randomized controlled trial to determine the effect of probiotics on postoperative nutritional indicators in liver cancer patients, which may provide a reference for adjuvant clinical nutrition support in this population.

## Materials and methods

### Participants

Patients with liver cancer (age ≥55, n=112) were collected from January 2021 to December 2022. The inclusion criteria: (1) no probiotics-related contraindications; (2) normal bowel function; (3) having received treatments of surgery, radiotherapy or immunotherapy; (4) normal consciousness level and voluntary participation. The exclusion criteria: (1) abnormal function of heart, kidney and other organs; (2) use of related-probiotics before intervening 3 months; (3) not cooperating with the research.

### Random allocation

**Figure 1:**
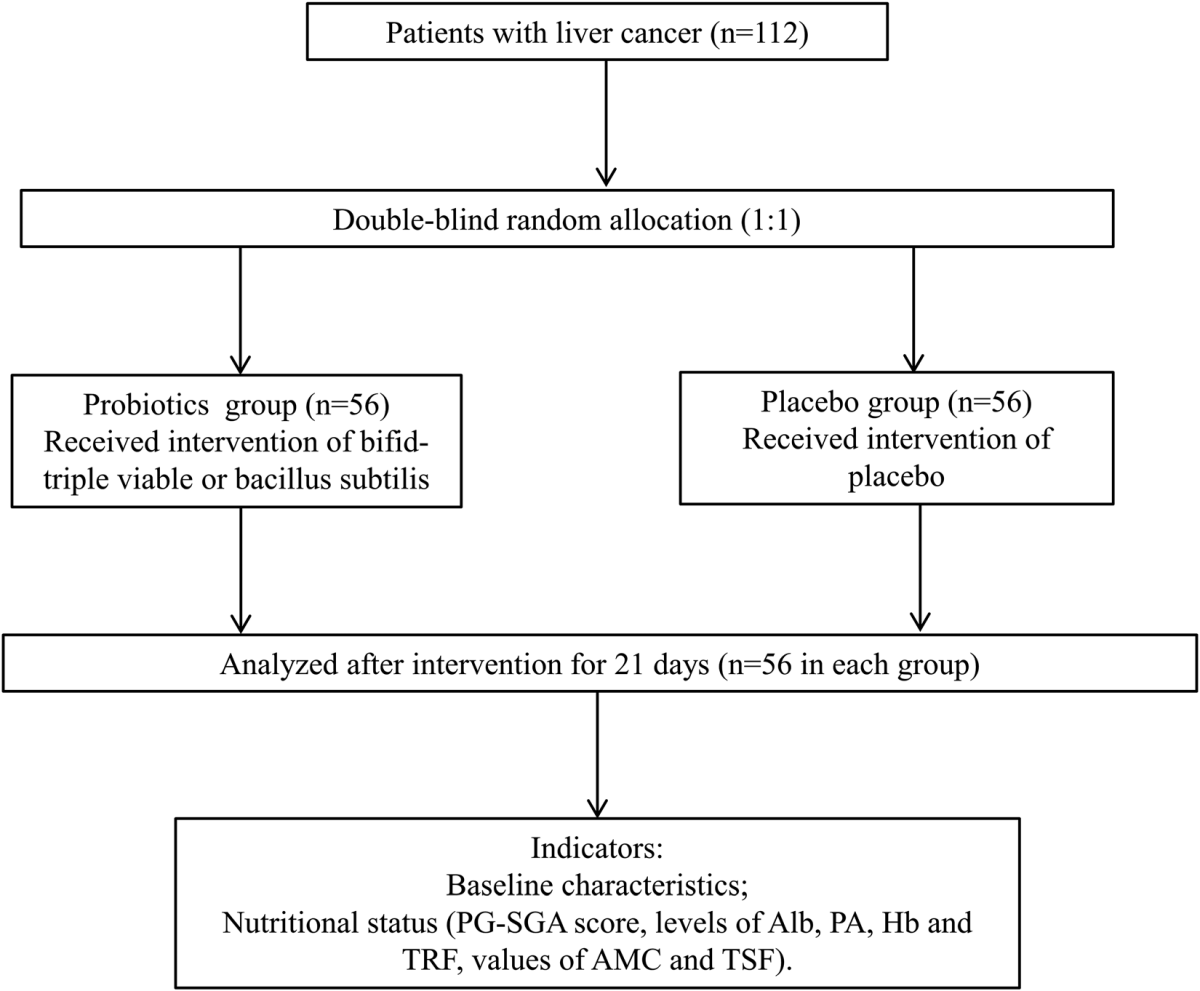
Flowchart diagram of the study.

Collected patients with liver cancer (n=112) were distributed into two groups (probiotics group and placebo group) according to different intervention ways (Figure 1). The allocation of patients was a double-blind random allocation method with the distribution ratio of 1:1 (56 patients in each group). The random distribution information of each patient was sealed in an opaque envelope, which was kept by a third party except doctors and nurses. The envelope corresponded to the registration number of each patient one by one, which was assigned to the patient after confirming qualification. Finally, after different interventions 7, 14 and 21 days (d), further analysis was performed between probiotics group (n=56) and placebo group (n=56).

### Description of intervention

Patients in both groups were given routine nursing (anti-cancer drugs, exercise guidance and careful observation) and nutritional support (diet guidance) during the hospitalization period. According to prior reports, interventions of probiotics and placebo were severally performed in probiotics group and placebo group [20], 21]. The probiotics group was given bifid-triple viable (Peifeikang, Shanghai Xinyi Pharmaceutical Inc., China; three times/d, 420 mg/time) or bacillus subtilis (Peifeikang, China Resources Zizhu Pharmaceutical Inc., China; three times/d, 0.5 g/time). The placebo group was given placebo. Both probiotics and placebo were supplied in capsules, which were completely same by their external appearance. The interventions of probiotics and placebo were continuously maintained for 21 d. During the intervention period, the diet was same in both groups.

### Observational indicators

#### Baseline characteristics

The basic clinical characteristics of patients in the two groups were accurately recorded, including gender, age, body mass index (BMI), nutritional risk screening (NRS 2002) score, surgery, radiotherapy, immunotherapy, cancer staging and probiotic species.

#### Patient generated subjective global assessment (PG-SGA) score

PG-SGA score was used to assess nutritional status between probiotics group and placebo group at different time points (after oral probiotics or placebo 0, 7, 14 and 21 d). According to the subjective nutrition assessment for cancer patients (WS/T 555–2017), seven aspects were assessed by PG-SGA, which were body weight, dietary intake, symptom, activity and function, disease status, metabolic requirement and physical examination. Then, they were categorized into four scores (A, B, C and D score). PG-SGA score=A score + B score + C score + D score. The higher PG-SGA score represented more poor nutritional status. PG-SGA score ≥9: patient was urgently needed to receive nutritional support [22], 23].

Dietary intake was assessed using two components: (1) change in intake over the past month: No change or greater than normal (0 points); Less than normal (1 point). (2) Current intake type: Normal diet (0 points), Normal diet but less than normal (1 point), Little solid food (2 points), Only liquids (3 points), Only nutritional supplements (3 points), Very little of anything (4 points). Tube feeding or intravenous nutrition only (0 points). The higher the score, the more severe the reduction in dietary intake.

Activity and function (physical activity) was assessed by asking: “How would you describe your activity level over the past month?” Options and scores: Normal with no limitations (0 points); Not quite normal but able to be up and about with fairly normal activities (1 point); Not feeling up to most things, but in bed or sitting less than half the day (2 points); Able to do little activity and spend most of the day in bed or sitting (3 points); Bedridden, rarely out of bed (4 points).

Metabolic requirement was assessed by evaluating the presence and severity of metabolic stress factors, including fever and use of glucocorticoids. Scoring: No metabolic stress (0 points); Mild metabolic stress (1 point); Moderate metabolic stress (2 points); Severe metabolic stress (3 points).

#### Quantitative dietary nutrient intake assessment

A detailed dietary survey was conducted to quantitatively assess baseline intake and monitor changes in nutrient consumption. The assessment was performed by uniformly trained clinical dietitians at two time points: after enrollment but before the intervention (baseline, day 0), and at the end of the 21-day intervention period (day 21). Dietary intake was evaluated using the 24-hour dietary recall for 3 consecutive days (including one weekend day) to estimate habitual intake. During structured interviews, patients reported all foods and beverages consumed. To enhance the accuracy of portion size estimation, a set of standardized food models (e.g., bowls, cups, spoons) and food portion picture aids were utilized. All reported dietary data were then entered and analyzed using the Nutritionist Pro software (version 1.5, First Databank, Inc., San Bruno, CA, USA) based on the Chinese Food Composition Table (Standard Edition). The primary outcomes derived from this analysis were each patient’s average daily intake of energy (kcal), protein (g), fat (g), carbohydrate (g).

#### The levels of albumin (Alb), prealbumin (PA), hemoglobin (Hb) and transferrin (TRF) in serums

The levels of Alb, PA, Hb and TRF in serums were used to evaluate nutritional status between probiotics group and placebo group. Before oral probiotics/placebo and after oral probiotics/placebo 7, 14 and 21 d, fasting venous blood was collected from each patient in the morning. After centrifuging, serums were obtained. Afterward, the Alb, PA, Hb and TRF were tested with a fully-auto specific protein analyzer (PA120, Genrui Biotech Inc., China), which were performed in accordance with manufacturer’ instruction. The increases of Alb, PA, Hb and TRF represented the improvement of nutritional status after intervention [20].

#### The values of arm muscle circumference (AMC) and triceps skinfold thickness (TSF)

The values of AMC and TSF were used to evaluate nutritional status between probiotics group and placebo group at different time points (after oral probiotics or placebo 0, 7, 14 and 21 d). According to early report, arm circumference value was measured in the middle part of the upper arm by an inextensible tape as well as TSF value was measured by a constant pressure lipocalibrator (Holtain Itd., UK), which were performed with three measurements. AMC value was calculated as arm circumference value minus π times TSF [21]. The increases of AMC and TSF represented the improvement of nutritional status after intervention [21].

#### Safety assessment and adverse events monitoring

Safety and tolerability were monitored throughout the study. Adverse events were assessed at each follow-up visit (days 7, 14, 21) via (1) patient self-reporting, (2) structured interviews, and (3) clinical measurements.

Nausea and vomiting: Actively inquired during interviews, with severity documented. Hair Loss: Assessed through direct patient questioning regarding increased shedding. Blood Pressure: Measured in duplicate using a calibrated electronic sphygmomanometer. Fluctuation Definition: Systolic blood pressure <90 mmHg or >140 mmHg, or diastolic blood pressure <60 mmHg or >90 mmHg, or a change of >20 mmHg in systolic or >10 mmHg in diastolic pressure from baseline.

Laboratory tests: Fasting venous blood was collected at baseline and day 21. white blood cell count: Analyzed from ethylene diamine tetraacetic acid (EDTA)-treated samples using an automated hematology analyzer (BC-760 CS, Mindray, Shenzhen, China). Leukopenia (<4.0 × 10^9^/L) or leukocytosis (>10.0 × 10^9^/L) was deemed abnormal. Hepatic and Renal Function: Serum from coagulant-treated samples was analyzed for alanine aminotransferase (ALT), aspartate aminotransferase (AST), creatinine (Cr), and blood urea nitrogen (BUN) on an automated biochemical analyzer (BS-1,000 M, Mindray, Shenzhen, China). Values exceeding standard reference ranges (e.g., ALT/AST>40 U/L; Cr >104/84 μmol/L for M/F; BUN >7.1 mmol/L) were considered abnormal.

### Evaluation of sample size

Sample size was analyzed by PASS 2021 software (NCSS Inc., USA). According to early research, the main result was set by the AMC value after intervening by probiotics for 21 d [24]. The number of patients in both groups was 1:1. It was assumed that AMC value in probiotics group was 19.58 ± 1.85 mm and that of in placebo group was 18.65 ± 1.33 mm. The power was set as 80 % and the type I error was set as 0.05. Sample drop-off rate was set as 10 %. Then, the required sample size of each group was calculated to be 52. Therefore, the included number of patients in each group (n=56) was effective.

### Statistical analysis

Continuity variables were described by mean ± standard deviation ( $\overset{®}{x}\;\pm \;s$ ) and calculated by SPSS 20.0 software (SPSS Inc., USA). The classification variables were compared by *χ* ^*2*^ test. Independent sample t test was used for comparison between the two groups. Repeated measurement-ANOVA was used for comparison at different time points in the same group. The level of significance was bilateral p<0.05.

### Design and ethic statement

This trial was a randomized, double-blind, placebo-controlled clinical trial, which was mainly designed in accordance with previous randomized controlled trials [25] and was conducted in accordance with the ethical principles of the Declaration of Helsinki. This research was authorized by the Ethical Committee of the Henan Vocational College of Nursing (Approval No.: KY202101-2). Informed consent was signed by each participant.

## Results

### Comparison of baseline characteristics

The mean age of the probiotics group (n=56, including 32 males and 24 females) was 63.57 ± 5.46 years and that of the placebo group (n=56, including 35 males and 21 females) was 62.36 ± 5.73 (Table 1). Furthermore, the mean BMI was 18.02 ± 2.04 in probiotics group and 17.86 ± 2.74 in placebo group (Table 1). As shown in Table 1, there were non-significant differences between probiotics group and placebo group about gender (p=0.563), age (p=0.253), BMI (p=0.727), NRS 2002 score (p=0.581), surgery (p=0.371), radiotherapy (p=0.246), immunotherapy (p=0.836), cancer staging (p=0.705), energy (p=0.193), protein (p=0.103), fat (p=0.169), carbohydrate (p=0.231). There was no tumor metastasis during this trial.

**Table 1:** Baseline characteristics ( $\overset{®}{x}\pm s$ ).

Indicator		Probiotics, n=56	Placebo, n=56	*t/χ* ^2^	p-Value
Gender, n	Male	32	35	0.334	0.563
	Female	24	21		
Age, year		63.57 ± 5.46	62.36 ± 5.73	1.149	0.253
BMI, kg/m^2^		18.02 ± 2.04	17.86 ± 2.74	0.351	0.727
NRS 2002 (score)		4.16 ± 0.87	4.25 ± 0.84	0.554	0.581
Surgery, n	Yes	41	45	0.801	0.371
	No	15	11		
Radiotherapy, n	Yes	37	31	1.348	0.246
	No	19	25		
Immunotherapy, n	Yes	40	39	0.043	0.836
	No	16	17		
Cancer staging, n	II	29	31	0.144	0.705
	III	27	25		
					
					
	Energy (kCal/d)	1,496.32 ± 22.79	1,502.44 ± 26.48	1.311	0.193
Dietary nutrient intake	Protein (g/d)	50.06 ± 4.25	48.72 ± 4.36	1.645	0.103
	Fat (g/d)	22.59 ± 2.41	23.24 ± 2.56	1.385	0.169
	Carbohydrate (g/d)	335.47 ± 18.64	331.54 ± 15.79	1.204	0.231

BMI, body mass index; NRS 2002, nutritional risk screening.

### Comparison of the PG-SGA score

**Figure 2:**
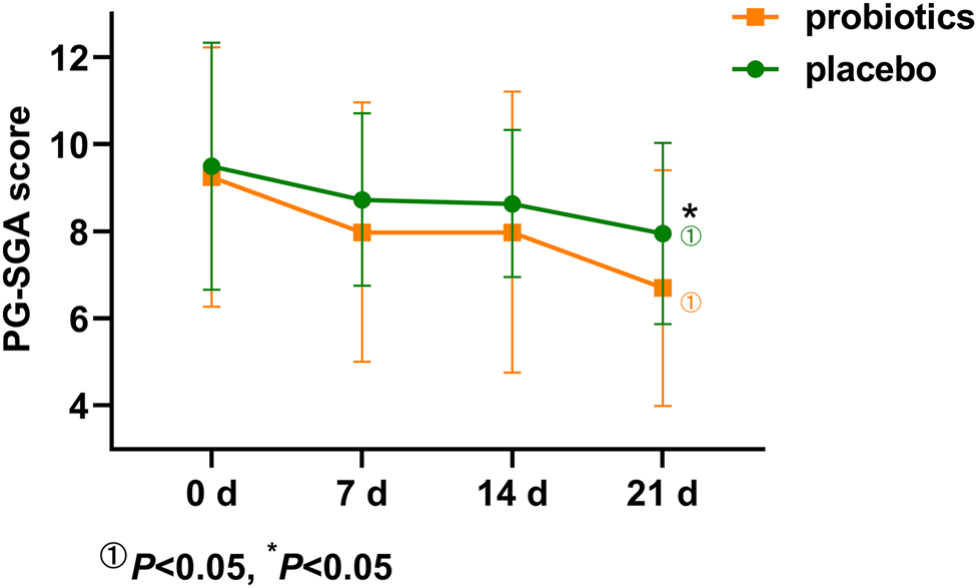
Comparison of the PG-SGA score. ^①^p<0.05 vs. Before oral administration/0 d; ^*^p<0.05 vs. probiotics group. PG-SGA, patient generated subjective global assessment; d, day.

After 21 d, it could be seen that PG-SGA score was obviously reduced in probiotics group (Figure 2, Table2, p*<*0.05) and placebo group (Figure 2, Table2 **,** p*<*0.05) relative to 0 d. Moreover, the decrease of PG-SGA score was more in probiotics group than placebo group (Figure 2, Table2, p*<*0.05).

**Table 2:** Comparison of the PG-SGA score ( $\overset{®}{x}\;\pm \;s$ ).

Group	n	PG-SGAscore			
		0 d	7 d	14 d	21 d
Probiotics group	56	9.25 ± 2.98	7.98 ± 2.98	7.98 ± 3.23	6.70 ± 2.71^ab^
Placebo group	56	9.50 ± 2.84	8.73 ± 1.98	8.64 ± 1.69	7.95 ± 2.08^a^

Compared with 0 d: ^a^p<0.05; compared with placebo group: ^b^p<0.05.

### Comparison of the levels of Alb, PA, Hb and TRF in serums

**Figure 3:**
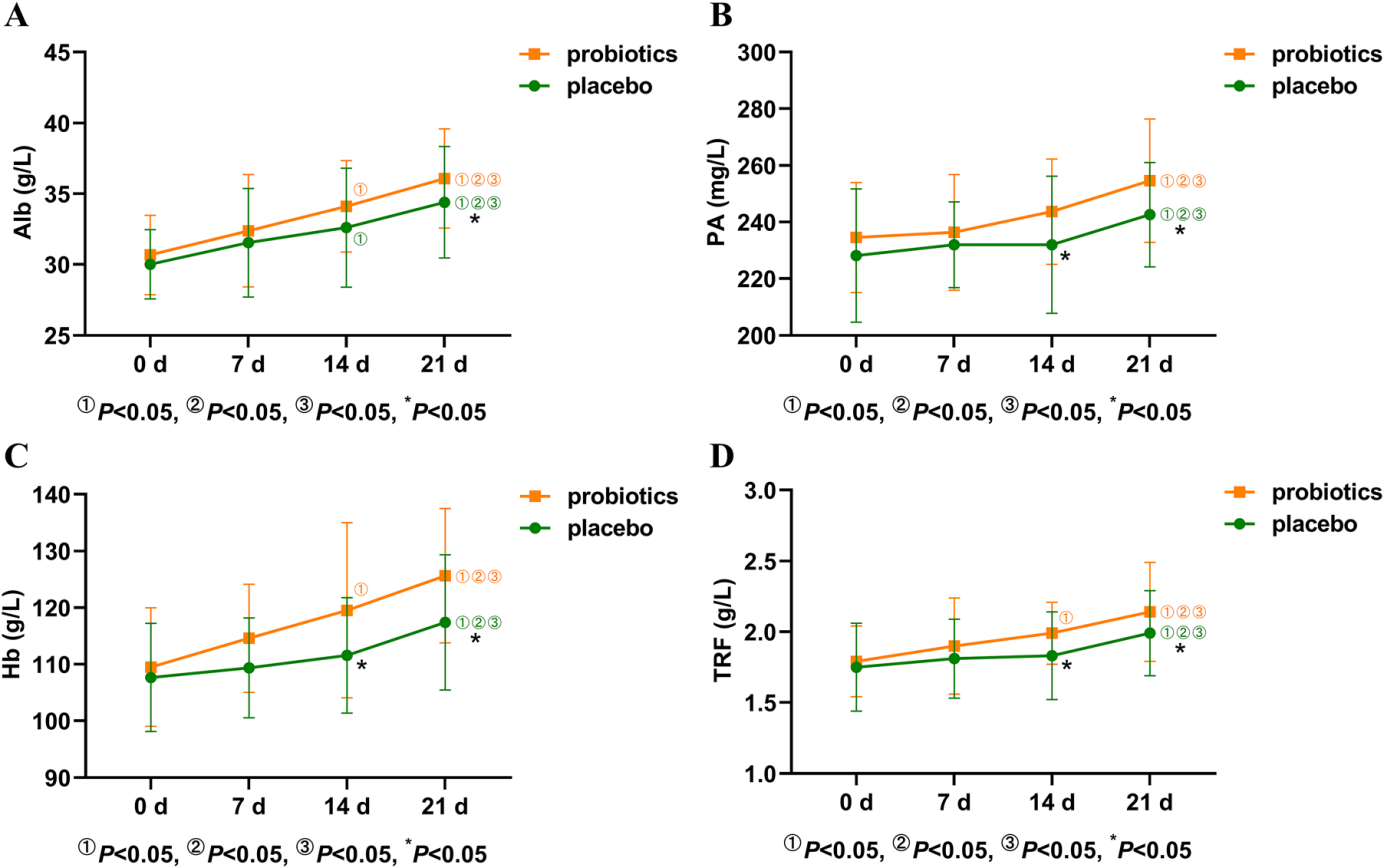
Comparison of the levels of Alb, PA, Hb and TRF in serums. (A-D) The levels of Alb (A), PA (B), Hb (C) and TRF (D) in serums were assessed at different time points (after oral probiotics or placebo 0, 7, 14 and 21 d). ^①^p<0.05 vs. Before oral administration/0 d; ^②^p<0.05 vs. 7 d; ^③^p<0.05 vs. 14 d; ^*^p<0.05 vs. probiotics group. Alb, albumin; PA, prealbumin; Hb, hemoglobin; TRF, transferrin; d, day.

As shown in Figure 3A–D and Table 3, the levels of Alb, PA, Hb and TRF in serums were assessed at different time points (after oral probiotics or placebo 0, 7, 14 and 21 d). After 14 d, the level of Alb in serums was significantly increased in probiotics group and placebo group compared to 0 d (Figure 3A, Table 3, p*<*0.05). After 14 d, the levels of Hb and TRF in serums were significantly up-regulated in probiotics group compared to 0 d (Figure 3C–D, Table 3, p*<*0.05). After 14 d, the increased levels of PA, Hb and TRF in serums were more in probiotics group than placebo group (Figure 3B–D, Table 3, p*<*0.05).

**Table 3:** Comparison of the levels of Alb, PA, Hb and TRF in serums ( $\overset{®}{x}\pm s$ ).

Group		Probiotics group	Placebo group
n		56	56
Alb, g/L	0 d	30.67 ± 2.81	30.01 ± 2.45
	7 d	32.38 ± 3.98	31.54 ± 3.84
	14 d	34.11 ± 3.23^a^	32.60 ± 4.21^a^
	21 d	36.08 ± 3.51^abcd^	34.40 ± 3.94^abc^
PA, mg/L	0 d	234.51 ± 19.45	228.16 ± 23.58
	7 d	236.35 ± 20.45	231.96 ± 15.16
	14 d	243.68 ± 18.65^d^	231.99 ± 24.25
	21 d	254.64 ± 21.81^abcd^	242.61 ± 18.47^abc^
Hb, g/L	0 d	109.48 ± 10.48	107.66 ± 9.56
	7 d	114.59 ± 9.56	109.35 ± 8.82
	14 d	119.51 ± 15.46^ad^	111.56 ± 10.21
	21 d	125.64 ± 11.85^abcd^	117.40 ± 11.95^abc^
TRF, g/L	0 d	1.79 ± 0.25	1.75 ± 0.31
	7 d	1.90 ± 0.34	1.81 ± 0.28
	14 d	1.99 ± 0.22^ad^	1.83 ± 0.31
	21 d	2.14 ± 0.35^abcd^	1.99 ± 0.30^abc^

Compared with pre-administration /0 d: ^a^p<0.05; compared with 7 d: ^b^p<0.05; compared with 14 d: ^c^p<0.05; compared with placebo group: ^d^p<0.05.

What’s more, after 21 d, the levels of Alb, PA, Hb and TRF in serums were elevated in probiotics group and placebo group compared to 0, 7 or 14 d (Figure 3A–D, Table 3, p*<*0.05). After 21 d, the increased levels of Alb, PA, Hb and TRF in serums were more in probiotics group than placebo group (Figure 3A–D, Table 3, p*<*0.05).

### Comparison of the values of AMC and TSF

**Figure 4:**
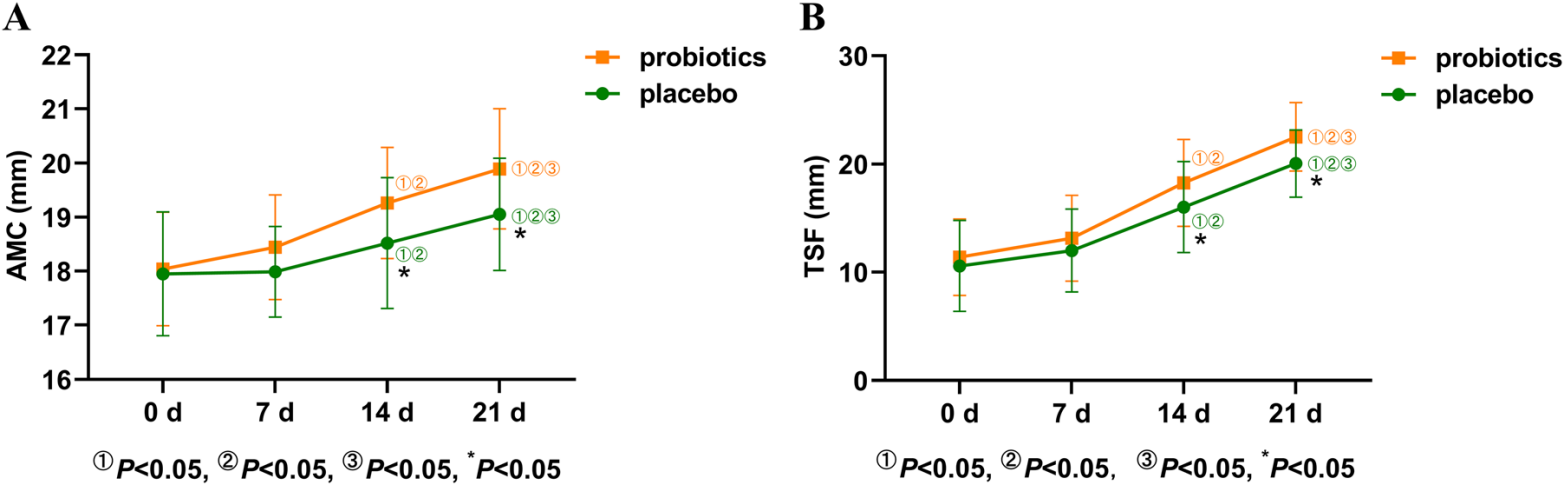
Comparison of the values of AMC and TSF. (A-B) The values of AMC (A) and TSF (B) at different time points (after oral probiotics or placebo 0, 7, 14 and 21 d) were assessed. ^①^p<0.05 vs. Before oral administration/0 d; ^②^p<0.05 vs. 7 d; ^③^p<0.05 vs. 14 d; ^*^p<0.05 vs. probiotics group. AMC, arm muscle circumference; TSF, triceps skinfold thickness; d, day.

Our outcomes revealed that there were increased trends of AMC value and TSF value in probiotics group and placebo group after 14 d or 21 d relative to 0 d (Figure 4A–B, Table 4, p*<*0.05). In addition, the increases of AMC value and TSF value were more in probiotics group than placebo group after 14 d or 21 d (Figure 4A–B, Table 4, p*<*0.05).

**Table 4:** Comparison of the values of AMC and TSF ( $\overset{®}{x}\pm s$ ).

Group		Probiotics group	Placebo group
n		56	56
AMC, mm	0 d	18.04 ± 1.05	17.95 ± 1.15
	7 d	18.44 ± 0.97	17.99 ± 0.84
	14 d	19.26 ± 1.0^abd^	18.52 ± 1.2^ab^
	21 d	19.89 ± 1.11^abcd^	19.05 ± 1.04^abc^
TSF, mm	0 d	11.38 ± 3.54	10.57 ± 4.21
	7 d	13.14 ± 3.97	11.99 ± 3.84
	14 d	18.26 ± 4.0^abd^	16.02 ± 4.2^ab^
	21 d	22.51 ± 3.15^abcd^	20.05 ± 3.11^abc^

Compared with pre-administration/0 d: ^a^p<0.05; compared with 7 d: ^b^p<0.05; compared with 14 d: ^c^p<0.05; compared with placebo group: ^d^p<0.05.

### Adverse reactions

The overall and specific incidences of adverse events were not significantly different between the probiotics and placebo groups (Overall incidence: 23.21 vs. 32.14 %, Table 5, p=0.291), indicating that the probiotic intervention was well-tolerated.

**Table 5:** Comparison of the incidence of adverse reactions between the two groups [cases (%)].

Group	Probiotics group	Placebo group	*χ* ^2^	p-Value
n	56	56		
Nausea and vomiting	2 (3.57)	4 (7.14)		
Hair loss	2 (3.57)	2 (3.57)		
Abnormal white blood cells	1 (1.79)	3 (5.36)		
Blood pressure fluctuation	3 (5.36)	5 (8.93)		
Abnormalities of the liver and kidneys	5 (8.93)	4 (7.14)		
Overall incidence rate, %	23.21	32.14	1.115	0.291

## Discussion

In recent years, there are more and more studies about the role of probiotics in improving nutrition level of cancer patients. A good nutrition status is beneficial to improve poor prognosis in liver cancer patients [26], 27]. In present study, our outcomes revealed that oral probiotics had a good effect on improving postoperative nutrition level in patients with liver cancer, indicating the potential utility of probiotics for improving poor nutrition in liver cancer patients.

Our findings are consistent with a growing body of evidence supporting the nutritional benefits of probiotics in oncology. For instance, after the use of probiotics, the postoperative rehabilitation process of GC patients was significantly shortened compared to patients treated with conventional enteral nutrition suspension, the immune function and nutritional status were validly improved and the prognostic survival rate was increased [28]. Supplementation of probiotics can reduce the incidence of gastrointestinal complications and improve the nutritional status of patients after surgery by regulating gastrointestinal flora, improving microecological balance and restoring the physiological function of gastrointestinal tract in esophageal cancer [29]. Similar positive effects have been reported in patients with colorectal cancer, where perioperative probiotic administration improved serum albumin and prealbumin levels and reduced postoperative inflammatory responses [30]. The consistency across these studies, despite differences in cancer types, underscores the potential broad applicability of probiotics in counteracting cancer-related malnutrition. Crucially, in our trial, the baseline characteristics, including quantitative dietary intakes of energy, protein, fat, and carbohydrate, were well-balanced between the probiotics and placebo groups. This comparability at baseline strengthens the validity of attributing the subsequent observed improvements primarily to the probiotic intervention rather than to pre-existing differences in nutritional intake.

Existing reports have elucidated that probiotics can exert its effect on anticancer, modulation of gut microbiota and immunomodulation [31], 32]. Kantapich Srikham et al. had revealed that the probiotic strains of streptococcus salivarius BP8 and BP160 can play their role in inhibiting the growth of liver cancer cells, suggesting probiotics has an underlying application for treating liver cancer disease [33]. The underlying mechanisms of probiotics are regulation of gut microbiota, improvement of intestinal injury, production of beneficial metabolites and increase of nutrients’ digestion and absorption [10], 15]. More importantly, probiotics can improve intestinal absorption function by reducing inflammation levels and then elevates nutrition level in host [34]. Combined with these findings, it is a speculation that probiotics can play a role in improving postoperative nutrition level in liver cancer patients, which is proved in this study.

To our knowledge, PG-SGA score is a classic tool to assess the nutritional statuses of patients with cancer. The decrease of PG-SGA score represents the improvement of nutritional status after nutrition intervention [35], 36]. Previous study showed that significant differences in the microbiota between individuals with malnutrition assessed by the PG-SGA [37]. Therefore, the effect of probiotics on nutritional statuses in postoperative patients with liver cancer was analyzed in this study. After 21 d, our outcome showed that PG-SGA score was reduced more in patients with probiotics than that in patients with placebo, suggesting probiotics had a role in improving nutritional status.

In addition, there are some common serum biochemical indicators (such as Alb, PA, Hb and TRF) and fat content detections (such as AMC and TSF) to assess nutritional statuses of patients with cancer. In the case of malnutrition, the contents of Alb, PA, Hb and TRF in serums can show a low level, which can be used to monitor the effect of nutrition intervention [20], 38]. AMC is an effective index to detect the whole body fat content and judge the subcutaneous fat development, which is a necessary item for nutrition survey [39]. TSF can calculate the body fat reserve and consumption and can indirectly reflect the change of energy [40]. The increases of Alb, PA, Hb, TRF, AMC and TSF represent the improvement of nutritional status after nutrition intervention. We found that the levels of Alb, PA, Hb and TRF as well as the values of AMC and TSF after 21 d were elevated more in patients with probiotics than that in patients with placebo, indicating probiotics had an influence on improving nutritional status. The concerted improvement across this comprehensive panel of markers, from rapid-turnover proteins like PA to anthropometric measurements like AMC, provides a robust and multi-faceted confirmation of the nutritional benefits. This pattern of improvement is consistent with that reported by Yang et al., who found significant changes in total protein (TP), PA, ALb and TRF after probiotic use in gastric cancer patients [41]. The reason may be that while maintaining the balance of intestinal flora, probiotics can also improve gastrointestinal blood supply, boost the recovery of absorption function, and promote the absorption and utilization of intestinal nutrients, which need further study.

## Limitations

Here are several limitations: (1) there is no long-term follow-up (more than 3 months) after oral probiotics on postoperative nutrition level; (2) it is unclear the effect of oral probiotics on quality of life; (3) it is unknown the effect of oral probiotics on nutrition level during perioperative period. (4) the probiotic intervention comprised two different formulations administered based on clinical practicality. While this approach validly addresses the primary question regarding the general utility of probiotics, it precludes definitive conclusions on the comparative efficacy of the specific strains used. Future studies with larger sample sizes and multi-group designs are needed to determine which specific type or combination of probiotics is most beneficial for postoperative nutritional support in patients with liver cancer.

## Conclusions

In sum, we found that oral probiotics had a good feedback on improving postoperative nutrition level in patients with liver cancer, which provided a theoretical basis for clinical application. After nutrition intervention 21 d, patients with probiotics displayed lower PG-SGA score than patients with placebo. Besides, after nutrition intervention 21 d, there were more obvious improvements of nutritional statuses in patients with probiotics than patients with placebo, which were manifested as increased levels of Alb, PA, Hb and TRF as well as values of AMC and TSF. For next investigations, a larger sample and various indicators will be explored to support above findings.

## Supplementary Material

Supplementary Material

Supplementary Material

## References

[1] Li Y, Zhang R, Xu Z, Wang Z (2022). Advances in nanoliposomes for the diagnosis and treatment of liver cancer. Int J Nanomed.

[2] Kihn-Alarcón AJ, Toledo-Ponce MF, Velarde A, Xu X (2019). Liver cancer in Guatemala: an analysis of mortality and incidence trends from 2012 to 2016. J Glob Oncol.

[3] Yang WS, Zeng XF, Liu ZN, Zhao QH, Tan YT, Gao J (2020). Diet and liver cancer risk: a narrative review of epidemiological evidence. Br J Nutr.

[4] Xue Y, Hu X, Wang D, Li D, Li Y, Wang F (2022). Gene editing in a Myo6 semi-dominant mouse model rescues auditory function. Mol Ther.

[5] Papadimitriou N, Markozannes G, Kanellopoulou A, Critselis E, Alhardan S, Karafousia V (2021). An umbrella review of the evidence associating diet and cancer risk at 11 anatomical sites. Nat Commun.

[6] Zhang HC, Wang LS, Miller E (2021). Hepatobiliary and pancreatic adverse events. Adv Exp Med Biol.

[7] Lee CC, Wu CY, Chen JT, Chen GH (2002). Comparing combined hepatocellular-cholangiocarcinoma and cholangiocarcinoma: a clinicopathological study. Hepatogastroenterology.

[8] Anwanwan D, Singh SK, Singh S, Saikam V, Singh R (2020). Challenges in liver cancer and possible treatment approaches. Biochim Biophys Acta Rev Cancer.

[9] Pinna AD, Yang T, Mazzaferro V, De Carlis L, Zhou J, Roayaie S (2018). Liver Transplantation and Hepatic Resection can Achieve Cure for Hepatocellular Carcinoma. Ann Surg.

[10] Qiao W, Yu F, Wu L, Li B, Zhou Y (2016). Surgical outcomes of hepatocellular carcinoma with biliary tumor thrombus: a systematic review. BMC Gastroenterol.

[11] Yan X, Liu L, Zhang Y, Song T, Liang Y, Liu Z (2021). Perioperative enteral nutrition improves postoperative recovery for patients with primary liver cancer: a randomized controlled clinical trial. Nutr Cancer.

[12] Wang L, Wang X, Wang X (2021). The effectiveness of enteral nutrition for patients with primary liver cancer: a randomized controlled study protocol. Medicine (Baltim).

[13] Park S, Park S, Lee SH, Suh B, Keam B, Kim TM (2016). Nutritional status in the era of target therapy: poor nutrition is a prognostic factor in non-small cell lung cancer with activating epidermal growth factor receptor mutations. Korean J Intern Med.

[14] Engelen M, Safar AM, Bartter T, Koeman F, Deutz NEP (2015). High anabolic potential of essential amino acid mixtures in advanced nonsmall cell lung cancer. Ann Oncol.

[15] Molska M, Reguła J (2019). Potential mechanisms of probiotics action in the prevention and treatment of colorectal cancer. Nutrients.

[16] So SS, Wan ML, El-Nezami H (2017). Probiotics-mediated suppression of cancer. Curr Opin Oncol.

[17] Samanta S (2022). Potential impacts of prebiotics and probiotics on cancer prevention. Anti Cancer Agents Med Chem.

[18] Javanmard A, Ashtari S, Sabet B, Davoodi SH, Rostami-Nejad M, Esmaeil Akbari M (2018). Probiotics and their role in gastrointestinal cancers prevention and treatment; an overview. Gastroenterol Hepatol Bed Bench..

[19] Zheng C, Chen T, Wang Y, Gao Y, Kong Y, Liu Z (2019). A randomised trial of probiotics to reduce severity of physiological and microbial disorders induced by partial gastrectomy for patients with gastric cancer. J Cancer.

[20] Ou Q, Wang L, Wang K, Shao P (2021). Effect of probiotics supplementation combined with WeChat platform health management on nutritional status, inflammatory factors, and quality of life in patients with mild-to-moderate ulcerative colitis: a randomized trial. Ann Palliat Med.

[21] Hevilla F, Padial M, Blanca M, Barril G, Jiménez-Salcedo T, Ramirez-Ortiz M (2023). Effect on nutritional status and biomarkers of inflammation and oxidation of an oral nutritional supplement (with or without probiotics) in malnourished hemodialysis patients. A multicenter randomized clinical trial “renacare trial. Front Nutr.

[22] Zhang Q, Li XR, Zhang X, Ding JS, Liu T, Qian L (2021). PG-SGA SF in nutrition assessment and survival prediction for elderly patients with cancer. BMC Geriatr.

[23] Kwang AY, Kandiah M (2010). Objective and subjective nutritional assessment of patients with cancer in palliative care. Am J Hosp Palliat Care.

[24] Caili Zhou GZ, Guo Q (2022). Effects of home enteral nutrition combined with probiotics on nutritional status of patients with esophageal cancer after radiotherapy. Electron J Metab Nutr Cancer (Chinese).

[25] Wauters L, Slaets H, De Paepe K, Ceulemans M, Wetzels S, Geboers K (2021). Efficacy and safety of spore-forming probiotics in the treatment of functional dyspepsia: a pilot randomised, double-blind, placebo-controlled trial. Lancet Gastroenterol Hepatol.

[26] Olveira G, González-Molero I (2016). An update on probiotics, prebiotics and symbiotics in clinical nutrition. Endocrinol Nutr.

[27] Wu H, Ganguly S, Tollefsbol TO (2022). Modulating microbiota as a new strategy for breast cancer prevention and treatment. Microorganisms.

[28] Liu Y, Jia M (2024). Shenqi injection plus probiotics improves nutritional status in patients with gastric cancer after partial gastrectomy. Pak J Pharm Sci.

[29] Liu C, Yang J, Dong W, Yuan J (2021). Effects of probiotics on gastrointestinal complications and nutritional status of postoperative patients with esophageal cancer: a protocol of randomized controlled trial. Medicine (Baltim).

[30] Mizuta M, Endo I, Yamamoto S, Inokawa H, Kubo M, Udaka T (2016). Perioperative supplementation with bifidobacteria improves postoperative nutritional recovery, inflammatory response, and fecal microbiota in patients undergoing colorectal surgery: a prospective, randomized clinical trial. Biosci Microbiota Food Health.

[31] Tiwari S, Dwivedi M, Rathod S, Bahadur P (2022). Immunomodulation and anticancer immunity: reviewing the potential of probiotics and their delivery with macromolecular carriers. Crit Rev Ther Drug Carrier Syst.

[32] Wan MLY, El-Nezami H (2018). Targeting gut microbiota in hepatocellular carcinoma: probiotics as a novel therapy. Hepatobiliary Surg Nutr.

[33] Srikham K, Daengprok W, Niamsup P, Thirabunyanon M (2021). Characterization of Streptococcus salivarius as new probiotics derived from human breast milk and their potential on proliferative inhibition of liver and breast cancer cells and antioxidant activity. Front Microbiol.

[34] Yang D, Meng XY, Wang Y, Zhang J, Zhao Y, Zheng ZC (2022). Effects of probiotics on gastric cancer-related inflammation: a systematic review and meta-analysis. J Food Biochem.

[35] Kim SH, Lee SM, Jeung HC, Lee IJ, Park JS, Song M (2019). The effect of nutrition intervention with oral nutritional supplements on pancreatic and bile duct cancer patients undergoing chemotherapy. Nutrients.

[36] van Dijk AM, Coppens BJP, van Beers MA, Bruins Slot AS, Verstraete CJR, de Bruijne J (2022). Nutritional status in patients with hepatocellular carcinoma: potential relevance for clinical outcome. Eur J Intern Med.

[37] Chao X, Lei Z, Hongqin L, Ziwei W, Dechuan L, Weidong D (2022). Faeces from malnourished colorectal cancer patients accelerate cancer progression. Clin Nutr.

[38] Wang L, Rui W, Chen S, Li Y, Ren M (2022). Effect of enteral and parenteral nutrition support on pulmonary function in elderly patients with chronic obstructive pulmonary disease complicated by respiratory failure. Comput Math Methods Med.

[39] Simpson F, Doig GS (2016). Bedside nutrition evaluation and physical assessment techniques in critical illness. Curr Opin Crit Care.

[40] Tang HK, Bowe SJ, Nguyen T, Dibley MJ (2020). Triceps and subscapular skinfold thickness percentiles of a school-based sample of adolescents in Ho Chi Minh City, Vietnam. Eur J Clin Nutr.

[41] Yang L, Liang Y, Li R, Chen Y, Zhang X (2024). Efficacy of probiotics combined with enteral nutrition therapy on intestinal flora, digestive tract symptoms and endogenous environment in patients with gastric cancer undergoing chemotherapy. Pak J Med Sci.

