# Supplementary material for: Effect of oral probiotics on postoperative nutrition level of patients with liver cancer
Source: Open Med (Wars). 2026 Aug 12;21(1):20261485. doi: 10.1515/med-2026-1485 (PMC13458991; doi:10.1515/med-2026-1485)
Supplement: Supplementary file 1 [file j_med-2026-1485_suppl_001.pdf]

ICS 11.020
C 55

WS

中 华 人 民 共 和 国 卫 生 行 业 标 准

WS/T 555—2017

肿瘤患者主观整体营养评估

Subjective nutrition assessment for cancer patients

2017－08－01 发布　　2018－02－01 实施

中华人民共和国国家卫生和计划生育委员会　发 布

前　　言

本标准按照GB/T 1.1-2009给出的规则起草。

本标准起草单位：中国抗癌协会、航空总医院、河北省人民医院、吉林大学第一医院、上海交通大学瑞金医院、黑龙江省肿瘤医院、昆明医科大学第一医院、四川大学华西医院、中山大学第一医院、厦门大学第一医院、天津市第三医院、中国医学科学院肿瘤医院、北京协和医院、天津市肿瘤医院、中南大学湘雅医院、广西医科大学肿瘤医院。

本标准主要起草人:石汉平、张晓伟、李薇、曹伟新、王晔、陈公琰、王昆华、伍晓汀、胡雯、石英英、罗琪、齐玉梅、吴健雄、丛明华、于康、巴一、陈子华、林源。

肿瘤患者主观整体营养评估

1 范围

本标准规定了对肿瘤患者进行营养评估的方法、范围、内容和结果判定等。

本标准适用于对已经确诊的尚未治疗和已经进行过治疗的恶性肿瘤患者进行营养评估，以确定其营养状况。

2 术语和定义

下列术语和定义适用于本文件。

2.1

营养评估 nutrition assessment

由受过培训的专业人员，采用营养评估工具，对患者的营养状况以及与营养相关的机体功能等进行全面检查和评价。

2.2

患者主观整体营养评估 patient-generated subjective nutrition assessment，PG-SGA

全面地收集主观资料（或信息），对患者营养状况进行评估。

2.3

营养不良 malnutrition

由能量、蛋白质及其他营养素不足或过剩造成的组织、形体和功能改变及相应的临床表现。

3 评估对象和方法

3.1 评估对象

评估对象应符合下列条件，即：年龄18岁以上的成年人，病理确诊为恶性肿瘤，神志清楚，无交流障碍，愿意接受评估，非濒临死亡。

3.2 评估时间

门诊的适用对象在其就诊时进行营养评估，住院的适用对象在其入院后48h内进行营养评估。家居肿瘤患者每3个月到门诊接受一次营养评估，住院肿瘤患者在一个治疗疗程结束后再次进行营养评估或每2周进行一次营养评估。

3.3 实施人员

受过培训的临床医师、临床营养师和护师。培训内容包括评估的程序、方法、内容、标准、结果判定及处理。实施人员应该有在他人（有评估经验者）指导下完成至少10例患者的评估经历，才能独立进行营养评估。

3.4 评估对象的告知

评估前应向评估对象简要介绍评估目的、内容及其必要性。如果营养评估是常规的诊治行为，则无需获得患者的知情同意。如果营养评估的目的是用于科学研究，则应获得患者的知情同意，并需要得到医院伦理委员会的批准。

4 评估内容及结果判断

4.1 评估内容

PG-SGA由患者自我评估及医务人员评估两部分组成，具体内容包括体重、进食情况、症状、活动和身体功能、合并疾病、应激、体格检查7个方面，前4个方面由患者自我评估，后3个方面由医务人员评估。

肿瘤患者主观整体营养评估记录表见附录A。

4.2 评分标准

见附录B。

4.3 结果判定

根据 PG-SGA 得分，将肿瘤患者的营养状况分为四类，见表 1。

表1 PG-SGA 分级评估结果判断

得分	评判结果
0 分～1 分	营养良好
2 分～3 分	可疑或轻度营养不良
4 分～8 分	中度营养不良
≥9 分	重度营养不良

4.4 营养干预

0分～1分：此时不需要干预措施，治疗期间保持常规随诊及评估。

2分～3分：由营养师、护师或医生进行患者或患者家庭教育，并可根据患者存在的症状和实验室检查的结果，进行药物干预。

4分～8分：由营养师进行干预，并可根据症状的严重程度，与医生、药师及护师联合进行营养干预。

≥9分：急需进行症状改善和/或同时进行营养干预。

附　录　A
（规范性附录）
肿瘤患者主观整体营养评估记录表

A.1　肿瘤患者主观整体营养评估记录表

采用 PG-SGA 对肿瘤患者进行营养评估时要实时记录，记录表见表 A.1。

表A.1　肿瘤患者主观整体营养评估记录表

<table>
<tr><th colspan="3">1 体重</th><th>2 进食情况</th></tr>
<tr><td>1 个月内体重下降率</td><td>评分</td><td>6 个月内体重下降率</td><td rowspan="5">在过去的一个月里，我的进食情况与平时情况相比：
□无变化（0）
□大于平常（0）
□小于平常（1）
我目前进食：
□正常饮食（0）
□正常饮食，但比正常情况少（1）
□进食少量固体食物（2）
□只能进食流质食物（3）
□只能口服营养制剂（3）
□几乎吃不下食物（4）
□只能依赖管饲或静脉营养（0）

第 2 项计分：__________</td></tr>
<tr><td>≥10%
5%～9.9%
3%～4.9%
2%～2.9%
0～1.9%</td><td>4
3
2
1
0</td><td>≥20%
10%～19.9%
6%～9.9%
2%～5.9%
0%～1.9%</td></tr>
<tr><td>2 周内体重无变化</td><td>0</td><td></td></tr>
<tr><td>2 周内体重下降</td><td>1</td><td></td></tr>
<tr><td>**第 1 项计分**</td><td colspan="2"></td></tr>
<tr><th colspan="3">3 症状</th><th>4 活动和身体功能</th></tr>
<tr><td colspan="3" rowspan="2">近 2 周来，我有以下的问题，影响我的饮食：
□ 没有饮食问题（0）
□ 恶心（1）　□ 口干（1）
□ 便秘（1）　□ 食物没有味道（1）
□ 食物气味不好（1）　□ 吃一会儿就饱了（1）
□ 其他（如抑郁、经济问题、牙齿问题）（1）
□ 口腔溃疡（2）　□ 吞咽困难（2）
□ 腹泻（3）　□ 呕吐（3）
□ 疼痛（部位）（3）
□ 没有食欲，不想吃饭（3）

第 3 项计分：__________</td><td>在过去的一个月，我的活动：
□ 正常，无限制（0）
□ 与平常相比稍差，但尚能正常活动（1）
□ 多数时候不想起床活动，但卧床或坐着时间不超过 12h（2）
□ 活动很少，一天多数时间卧床或坐着（3）
□ 几乎卧床不起，很少下床（3）

第 4 项计分：__________</td></tr>
<tr><td>**第 1～4 项计分（A 评分）：**__________</td></tr>
</table>

表 A.1 肿瘤患者主观整体营养评估记录表（续）

5. 合并疾病	
**疾病**	**评分**
肿瘤	1
艾滋病	1
呼吸或心脏疾病恶液质	1
存在开放性伤口或肠瘘或压疮	1
创伤	1
**年龄**	**评分**
超过 65 岁	1
**第 5 项计分（B 评分）**	

6. 应激				
**应激**	**无（0）**	**轻（1 分）**	**中（2 分）**	**重（3 分）**
发热	无	37.2～38.3 ℃	38.3～38.8 ℃	>38.8 ℃
发热持续时间	无	<72h	72h	>72h
是否用激素（强的松）	无	低剂量（<10mg/d 强的松或相当剂量的其它激素）	中剂量（10mg/d～30mg/d 强的松或相当剂量的其它激素）	大剂量（>30mg/d 强的松或相当剂量的其它激素）
**第 6 项计分（C 评分）**				

7. 体格检查				
**项目**	**0 分**	**1 分**	**2 分**	**3 分**
肌肉状况				
颞部（颞肌）				
锁骨部位（胸部三角肌）				
肩部（三角肌）				
肩胛部（背阔肌、斜方肌、三角肌）				
手背骨间肌				
大腿（四头肌）				
小腿（腓肠肌）				
总体肌肉丢失评分				
**第 7 项计分（D 评分）**				
**总分=A+B+C+D________**				

附　录　B
（规范性附录）
评分标准

B.1　病人自评表（A评分）

B.1.1　体重评分

体重评分见表B.1。

表B.1　病人自评表

目前我的体重约为　　kg<br>目前我的身高约为　　cm<br>1 个月前我的体重约为　　kg<br>6 个月前我的体重约为　　kg<br><br>最近两周内我的体重<br>无改变（0）<br>增加（0）<br>下降（1）
操作说明：<br>患者目前体重为实测体重。任何原因使患者不能自行测量体重时，可抱起患者一起测量，再测量并减去抱起人的体重。<br>1 个月前的体重和 6 个月前的体重患者可能记不清，此时，可采取在目前体重的基础上逐渐加量询问或逐渐减量询问，根据患者本人选定的近似值填写体重。例如，患者目前体重为 50kg，可以询问患者 1 个月前大约有 51kg、52kg、53kg、54kg、55kg，或 49kg、48kg、47kg、46kg、45kg，然后根据患者本人选定的数字，作为 1 个月前的体重。<br>体重下降百分率是指下降体重占原体重的百分比。例如患者 1 个月前体重 50kg，目前体重 46kg，1 个月内下降 4kg，则下降百分比为（50-46）/50=8%。<br>表 1 以 1 个月的体重变化情况评分，没有 1 个月体重变化资料时，则以 6 个月体重变化情况评分。2 周内体重下降需另记 1 分，无下降为 0 分。两者相加为体重总分。<br>无法准确了解具体体重时，可根据患者体重下降程度：无/轻/中/重/极重，自我评分为 0/1/2/3/4 分。

B.1.2　患者自评表各项计分方法

表 1 第 1 项计分方法：本项为累计计分。

表 1 第 2 项计分方法：本项为多选，但是计分不做累加，以最高分选项为本项计分。

表 1 第 3 项计分方法：本项症状为近 2 周内经常出现的症状，偶尔一次出现的症状不能作为选择，本项为多选，累计计分。如没有食欲、不想吃，记 3 分；恶心，记 1 分；呕吐，记 3 分；口腔溃疡，记 2 分；腹泻，记 3 分；该项最后得分为 3+1+3+2+3=12 分

表 1 第 4 项计分方法：本项为单选，取最符合的一项作为本项计分。

B.2　医务人员评估表

B.2.1　合并疾病（B评分）

合并疾病（B评分），见表B.2。

表B.2　合并疾病（B 评分）

**合并疾病及其与营养需求的关系**<br>相关诊断（详细说明）:<br>肿瘤分期：Ⅰ Ⅱ Ⅲ Ⅳ<br>其他<br>年龄
操作说明<br>按表1第5项做单项或多项选择，累计计分。如果患者存在表1第5项中没有列举出来的疾病，不予记分。B评分中的“其他”指分期不确定或不同分期体系。

B.2.2　应激（C评分）

应激（C评分），见表B.3。

表B.3　应激（C 评分）

目前体温____℃；<br>如果为发热，发热持续时间______h；<br>是否用糖皮质激素 □ 是 药名_____ 最大总剂量/天(mg/d)_____ □ 否
操作说明<br>患者体温为评估当时实测体温。这里的“发热”定义为本次调查时刻的体温升高，而不是病历体温单记录的体温升高。如果调查时体温升高，需了解此刻前 3 天的体温及激素使用情况。如果调查时刻体温不升高，即记录为无发热。<br>发热持续时间为本次发热已经持续的时间。<br>激素使用是指因为本次发热而使用的激素，如果连续多日使用不同剂量的激素，取其平均值作为激素剂量。其它原因如结缔组织病使用的激素，不作评估。<br>C 评分（见表 1 第 6 项）为累计评分。如患者体温 37.5℃，记 1 分；持续发热已经 4 天，记 3 分；每天使用 20mg 强的松，记 2 分。总记分为 6 分。

B.2.3　体格检查（D评分）

体格检查包括肌肉的7个方面。检查顺序是从上到下，从头到脚。先看颞肌，再往下到锁骨部位（胸部三角肌）、肩部（三角肌）、肩胛部（背阔肌、斜方肌、三角肌），再检查手背骨间肌肉（尤其是虎口处）；最后依次检查大腿（四头肌）、小腿（腓肠肌）。见表B.4。

表B.4　体格检查

项目	得分
肌肉	
操作说明<br>按多数部位情况确定患者肌肉得分，如多数部位肌肉为轻度丢失，则肌肉情况的最终得分即为轻度，记 1 分；如多数部位肌肉为中度丢失，则肌肉情况的最终得分为 2 分。	

B.3 体格检查评分标准

B.3.1 肌肉情况评估

肌肉情况评估见表B.5。

表B.5 肌肉情况评估

部位	检查要旨	0 分	1 分	2 分	3 分
颞部（颞肌）	直接观察,让患者头转向一边	看不到明显的凹陷	轻度凹陷	凹陷	显著凹陷
锁骨部位(胸部三角肌)	看锁骨是否凸出及其程度	青年男性看不到锁骨,女性及成年男性看到但不凸出	部分凸出	凸出	明显凸出
肩部（三角肌）	看肩部是否凸出，形状，手下垂	圆形	肩峰轻度凸出	介于二者之间	肩锁关节方形，骨骼凸出
骨间肌	观察手背，拇指和食指对捏，观察虎口处是否凹陷	拇指和食指对捏时肌肉凸出，女性可平坦	平坦	平坦和凹陷	明显凹陷
肩胛骨（背阔肌、斜方肌、三角肌）	患者双手前推，看肩胛骨是否凸出	肩胛骨不凸出，肩胛骨内测不凹陷	肩胛骨轻度凸出，肋、肩胛、肩、脊柱间轻度凹陷	肩胛骨凸出，肋、肩胛、肩、脊柱间凹陷	肩胛骨明显凸出，肋、肩胛、肩、脊柱间显著凹陷
大腿（股四头肌）		圆润，张力明显	轻度消瘦，肌肉较弱	介于二者之间	大腿明显消瘦，几乎无肌张力
小腿（腓肠肌）		肌肉发达	消瘦，有肌肉轮廓	消瘦，肌肉轮廓模糊	消瘦，无肌肉轮廓，肌肉松驰无力
肌肉消耗得分					
