# Supplementary material for: Effect of oral probiotics on postoperative nutrition level of patients with liver cancer
Source: Open Med (Wars). 2026 Aug 12;21(1):20261485. doi: 10.1515/med-2026-1485 (PMC13458991; doi:10.1515/med-2026-1485)
Supplement: Supplementary file 2 [file j_med-2026-1485_suppl_002.docx]

ICS 11.020 C 55

**WS**

Health Industry Standard of the People's Republic of China

WS/T 555—2017

Subjective nutrition assessment for cancer patients

2017 - 08 - 01 Issued	2018 - 02- 01 Implemented

Issued by the National Health and Family Planning Commission of the People's Republic of China

Foreword

This standard is drafted in accordance with the rules specified in GB/T 1.1-2009.

Drafting units of this standard: China Anti-Cancer Association, Aviation General Hospital, Hebei Provincial People's Hospital, The First Hospital of Jilin University, Ruijin Hospital Affiliated to Shanghai Jiao Tong University, Heilongjiang Provincial Cancer Hospital, The First Affiliated Hospital of Kunming Medical University, West China Hospital of Sichuan University, The First Affiliated Hospital of Sun Yat-sen University, The First Affiliated Hospital of Xiamen University, Tianjin Third Central Hospital, Cancer Hospital of the Chinese Academy of Medical Sciences, Peking Union Medical College Hospital, Tianjin Medical University Cancer Institute and Hospital, Xiangya Hospital of Central South University, and Guangxi Medical University Cancer Hospital.

Chief drafters of this standard: Hanping Shi, Xiaowei Zhang Wei, Li, Weixin Cao, Ye Wang, Gongyan Chen, Kunhua Wang, Xiaoting Wu, Wen Hu, Yingying Shi, Qi Luo, Yumei Qi, Jianxiong Wu, Minghua Cong, Kang Yu, Yi Ba, Zihua Chen, and Yuan Lin.

Subjective Global Nutritional Assessment for Cancer Patients

1 Scope

This standard specifies the methods, scope, content, and result determination for nutritional assessment of cancer patients.

This standard is applicable to the nutritional assessment of patients with confirmed malignant tumors who have not yet received treatment or have received treatment, so as to determine their nutritional status.

2 Terms and Definitions

The following terms and definitions apply to this document.

2.1 Nutrition assessment

A comprehensive examination and evaluation of a patient's nutritional status and nutrition-related bodily functions conducted by trained professionals using nutritional assessment tools.

2.2 Patient-Generated Subjective Global Assessment (PG-SGA)

A comprehensive collection of subjective data (or information) to assess a patient's nutritional status.

2.3 Malnutrition

Changes in tissue, body shape, and function, as well as corresponding clinical manifestations, caused by insufficient or excessive energy, protein, and other nutrients.

3 Assessment Subjects and Methods

3.1 Assessment Subjects

Subjects for assessment shall meet the following criteria: adults aged 18 years or above, pathologically confirmed malignant tumors, clear consciousness, no communication barriers, willingness to accept assessment, and not terminally ill.

3.2 Assessment Time

For applicable outpatients, nutritional assessment shall be conducted during their consultation; for applicable inpatients, nutritional assessment shall be conducted within 48 hours after admission. Home-dwelling cancer patients shall receive nutritional assessment at the outpatient department once every 3 months, and hospitalized cancer patients shall undergo nutritional assessment again after the end of a treatment course or once every 2 weeks.

3.3 Implementers

Trained clinical physicians, clinical dietitians, and nurses. The training content includes assessment procedures, methods, content, standards, result determination, and handling. Implementers shall have experience in completing assessments for at least 10 patients under the guidance of others (those with assessment experience) before they can conduct nutritional assessments independently.

3.4 Informing Assessment Subjects

Before the assessment, the purpose, content, and necessity of the assessment shall be briefly explained to the assessment subjects. If the nutritional assessment is a routine diagnosis and treatment behavior, it is not necessary to obtain the patient's informed consent. If the nutritional assessment is for scientific research purposes, the patient's informed consent shall be obtained, and approval from the hospital ethics committee shall be required.

4 Assessment Content and Result Determination

4.1 Assessment Content

PG-SGA consists of two parts: patient self-assessment and medical staff assessment. The specific content includes 7 aspects: body weight, eating status, symptoms, activity and physical function, comorbid diseases, stress, and physical examination. The first 4 aspects are assessed by the patient themselves, and the last 3 aspects are assessed by medical staff.

The Subjective Global Nutritional Assessment Record Form for Cancer Patients is shown in Appendix A.

4.2 Scoring Standards

See Appendix B.

4.3 Result Determination

Based on the PG-SGA score, the nutritional status of cancer patients is divided into four categories, as shown in Table 1.

Table 1 Judgment of PG-SGA Grading Assessment Results

| Score range | Evaluation result |
| --- | --- |
| 0 ～1 point | Well-nourished |
| 2 ～3 point | Suspected or mild malnutrition |
| 4 ～8 point | Moderate malnutrition |
| ≥9 point | Severe malnutrition |

4.4 Nutritional Intervention

0～1 points: No intervention measures are needed at this time; regular follow-up and assessment shall be maintained during treatment.

2～3 points: Patient or patient family education shall be conducted by dietitians, nurses, or doctors, and drug intervention may be carried out based on the patient's existing symptoms and laboratory test results.

4～8 points: Intervention shall be conducted by dietitians, and combined nutritional intervention with doctors, pharmacists, and nurses may be carried out according to the severity of symptoms.

≥9 points: Urgent symptom improvement and/or simultaneous nutritional intervention are required.

Appendix A

(Normative Appendix)

Subjective Global Nutritional Assessment Record Form for Cancer Patients

A.1 Subjective Global Nutritional Assessment Record Form for Cancer Patients

When conducting nutritional assessment for cancer patients using PG-SGA, real-time recording shall be performed, and the record form is shown in Table A.1.

Table A.1 Subjective Global Nutritional Assessment Record Form for Cancer Patients

| **1** **weight** | | | **2** **Eating situation** |
| --- | --- | --- | --- |
| Weight loss rate within 1 month | Score | Weight loss rate within 6 months | In the past month, compared with my usual situation, my eating status is:  □No change（0）  □More than usual（0）  □Less than usual  （1）My current eating is：  □Normal diet （0）  □Normal diet but less than normal amount（1）  □Eating a small amount of solid food（2）  □Only able to eat liquid food（3）  □Hardly able to eat any food（4）  □Only dependent on tube feeding or parenteral nutrition（0）  **Item 2 Scoring：** |
| ≥10%  5%～9.9%  3%～4.9%  2%～2.9%  0～1.9% | 4  3  2  1  0 | ≥20%  10%～19.9%  6%～9.9%  2%～5.9%  0%～1.9% |  |
| No weight change within 2 weeks | 0 |  |  |
| Weight loss within 2 weeks | 1 |  |  |
| **Item 1 Scoring** |  | |  |
| **3** Symptoms | | | **4** Activity and Physical Function |
| In the past 2 weeks, I have the following problems that affect my diet：  □ No dietary problems（0）  □ Nausea（1） □ Dry mouth（1）  □ Constipation（1） □ Food has no taste（1）  □ The food smells bad（1） □ feel full after eating for a while（1）  □ Others(such as depression, economic problems, dental problems)（1）  □ Oral ulcer（2） □ Difficulty in swallowing（2）  □ Diarrhea（3） □ Vomiting（3）  □ Pain (Location)（3）  □ Have no appetite and don't want to eat（3）  **Item 3 Scoring：** | | | In the past month, my activity is：  □ Normal, unrestricted（0）  □ It's slightly worse than usual, but still able to move normally（1）  □ Most of the time, I don't want to get up and move around, but I don't spend more than 12 hours in bed or sitting（2）  □ Very little activity, spending most of the day in bed or sitting（3）  □ Almost bedridden and rarely getting out of bed（3）  **Item 4 Scoring：** |
|  |  |  | **Item 1-4 Scoring（A** **score）：** |

Table A.1 Record Form of Subjective Holistic Nutritional Assessment for Cancer Patients (Continued)

| **5.** Comorbid Diseases | | | | | | | | | |
| --- | --- | --- | --- | --- | --- | --- | --- | --- | --- |
| Diseases | | | | | **score** | | | | |
| Tumor  AIDS  Cachexia due to respiratory or cardiac diseases  Presence of open wounds, intestinal fistulas, or pressure ulcers  Trauma | | | | | 1  1  1  1  1 | | | | |
| **Age** | | | | | **score** | | | | |
| Over 65 years old | | | | | 1 | | | | |
| **Item 5 Scoring（B** **score）** | | | | |  | | | | |
| **6.** stress | | | | | | | | | |
| stress | **No（0）** | | **Light（1** **score）** | | | **Medium（2** **score）** | | **Heavy（3** **score）** | |
| fever  Duration of fever  Whether to use hormones  （Prednisone） | No  No  No | | 37.2～38.3 ℃ <72h  Low dose (<10mg/d prednisone or equivalent dose of other hormones) | | | 38.3～38.8 ℃  72h  Medium dose (10mg/d to 30mg/d of prednisone or equivalent doses of other hormones) | | >38.8 ℃  >72h  High doses (>30mg/d of prednisone or equivalent doses of other hormones) | |
| **Item 6 Scoring（C** **score）** |  | | | | | | | | |
| **7.** Physical examination | | | | | | | | | |
| **Item** | | **0** **score** | | **1** **score** | | | **2** **score** | | **3** **score** |
| Muscle condition  Temporal region (temporal muscle)  The collarbone area (deltoid muscle of the chest)  Shoulder (deltoid muscle)  Scapular region (latissimus dorsi, trapezius, deltoid)  Interosseous muscles of the dorsal hand  Thigh (quadriceps)  Calf (gastrocnemius muscle)）  Overall muscle loss score | |  | |  | | |  | |  |
| **Item 7 Scoring（D** **score）** | |  | | | | | | | |
| **Total score=A+B+C+D________** | | | | | | | | | |

Appendix B

(Normative Appendix)

Scoring Standards

B.1 Patient Self-Assessment Form (Score A)

B.1.1 Body Weight Score

The body weight score is shown in Table B.1.

Table B.1 Patient Self-Assessment Form

My current weight is approximately	kg

My current height is approximately	cm

1 month ago,My weight was about	kg

6 month ago,My weight was about	kg

My weight in the past two weeks

No change（0）

Increase（0）

Decline（1）

Operating Instructions：

The patient's current weight is the measured weight. If for any reason the patient is unable to measure their weight by themselves, the patient can be picked up and measured together, then the weight of the person being picked up can be measured and subtracted.

The patient may not be able to remember their weight one month ago or six months ago. At this point, it is advisable to gradually increase or decrease the weight on the basis of the current weight and fill in the weight according to the approximate value selected by the patient themselves. For instance, if a patient's current weight is 50kg, you can ask the patient about their weight one month ago, which was approximately 51kg, 52kg, 53kg, 54kg, 55kg, or 49kg, 48kg, 47kg, 46kg, 45kg. Then, based on the number selected by the patient themselves, take it as their weight one month ago.

The percentage of weight loss refers to the proportion of weight lost to the original weight. For instance, if a patient weighed 50kg one month ago and currently weighs 46kg, and has lost 4kg within one month, then the percentage decrease is (50-46) /50=8%.

Table 1 scores the weight changes over one month. When there is no data on weight changes over one month, the weight changes over six months are used for scoring. If weight loss occurs within two weeks, an additional 1 point will be awarded; if there is no weight loss, it will be 0 points. The sum of the two is the total weight score.

When it is impossible to accurately determine the specific weight of the patient, the degree of weight loss can be considered as: none/light/moderate/heavy/extremely heavy, with a self-rating of 0/1/2/3/4 points.

B.1.2 The scoring methods for each item on the patient self-assessment form

Scoring method for Item 1 of Table 1: This item is a cumulative scoring.

Scoring method for Item 2 in Table 1: This item is multiple-choice, but the scores will not be accumulated. The highest score option will be used to score this item.

Scoring method for Item 3 in Table 1: This symptom refers to those that frequently occur within the past two weeks. Occasional symptoms cannot be selected. This item is a multiple-choice option and is scored cumulatively. If there is no appetite or no desire to eat, 3 points will be deducted. Disgusting, 1 point deducted; Vomiting, 3 points deducted; Oral ulcer, 2 points deducted; Diarrhea, 3 points deducted; The final score for this item is 3+1+3+2+3=12 points

Scoring method for Item 4 in Table 1: This item is a single-choice choice. The most suitable option will be taken as the score for this item.

B.2 Medical Staff Assessment Form

B.2.1 Comorbid Diseases（B score）

Comorbid Diseases (B score), as shown in Table B.2.

Table B.2 Comorbid Diseases(B score)

**Concurrent diseases and their relationship with nutritional requirements**

Relevant diagnosis (detailed description):

Tumor staging: Ⅰ, Ⅱ, Ⅲ, Ⅳ

Others

Age

Operating Instructions

Make single or multiple choices according to item 5 in Table 1 and accumulate the scores. If a patient has a disease not listed in item 5 of Table 1, no points will be awarded. In the B score, "other" refers to an uncertain stage or a different stage system.

B.2.2 Stress(C score)

Stress(C score), as shown in Table B.3。

Table B.3 Stress（C score）

Current body temperature____ ℃;

If it is fever, the duration of fever is______h；

Whether to use glucocorticoids □ Yes drug name_____ maximum total dose/day (mg/d)_____	□ No

Operating Instructions

The patient's body temperature was measured at the time of assessment. Here, "fever" is defined as the increase in body temperature at the time of this survey, rather than the increase in body temperature recorded on the medical record's temperature chart. If the body temperature rises during the investigation, it is necessary to understand the body temperature and hormone usage in the three days prior to this moment. If the body temperature does not rise at the time of the investigation, it is recorded as no fever.

The duration of the fever refers to the time that the current fever has persisted.

Hormone use refers to the use of hormones due to the current fever. If different doses of hormones are used continuously for several days, the average value is taken as the hormone dose. Other causes such as hormones used in connective tissue diseases are not evaluated.

The C score (see item 6 in Table 1) is the cumulative score. If the patient's body temperature is 37.5℃, 1 point will be deducted. If the fever has persisted for 4 days, 3 points will be deducted. 2 points will be awarded for using 20mg of prednisone daily. The total score is 6 points.

B.2.3 Physical examination (D score)

The physical examination includes seven aspects of the muscles. The inspection sequence is from top to bottom and from head to toe. First, examine the temporal muscle, then move down to the clavicle area (deltoid muscle of the chest), shoulder (deltoid muscle), scapular region (latissimus dorsi, trapezius, deltoid muscle), and finally check the interphalangeal muscles of the hand (especially at the tiger's mouth). Finally, check the thigh (quadriceps) and the calf (gastrocnemius) in sequence. See Table B.4.

Table B.4 Physical examination

| Items | Score |
| --- | --- |
| Muscle |  |
| Operating Instructions  The patient's muscle score is determined based on the condition of most parts. If the muscles in most parts are slightly lost, the final score of the muscle condition is mild and 1 point is recorded. If the muscles in most parts are moderately lost, the final score for the muscle condition is 2 points. | |

B.3 Physical examination scoring criteria

B.3.1 Muscle condition assessment

The assessment of muscle condition is shown in Table B.5.

Table B.5 Muscle condition assessment

| Part | Key points of inspection | 0 score | 1 score | 2 分 | 3 score |
| --- | --- | --- | --- | --- | --- |
| Temporal region (temporal muscle | Observe directly and turn the patient's head to one side | There is no obvious depression | Mild depression | Depression | Significant depression |
| The collarbone area (deltoid muscle of the chest) | Check whether the collarbone protrudes and to what extent | Young men cannot see their collarbones, while women and adult men can see them but they do not protrude | Partially protruding | Protruding | SignificantProtruding |
| Shoulder (deltoid muscle) | Check if the shoulders are protruding, the shape, and the hands are hanging down | Circle | The acromion is slightly protruding | Between the two | The acromioclavicular joint is square and the bone protrudes |
| Interosseous muscle | Observe the back of your hand, pinch your thumb and index finger together, and check if there is any depression at the thumb and index finger | When the thumb and index finger are pinched together, the muscles protrude. For women, it can be flattened | Flat | Flat and depression | Obvious depression |
| Scapula (latissimus dorsi, trapezius, deltoid) | The patient pushes both hands forward to see if the shoulder blades protrude | The scapula does not protrude, and the inner part of the scapula does not depression | The scapula is slightly protruding, and there is a slight depression between the ribs, scapula, shoulders and spine | The scapula protrudes, and the ribs, scapula, shoulders and spine are concave | The scapula is prominently protruding, and there are significant depressions between the ribs, scapula, shoulders and spine |
| Thigh (quadriceps) |  | Smooth and round, with obvious tension | Mild emaciation and weak muscles | Between the two | The thighs are obviously thin and have almost no muscle tone |
| Calf (gastrocnemius muscle |  | Well-developed muscles | Thin, with muscular contours | Emaciated, with indistinct muscle contours | Emaciated, with no muscle contours, and loose and weak muscles |
| Muscle expenditure score |  | | | | |
